# Classification and function of γδT cells and its research progress in anti-glioblastoma

**DOI:** 10.1007/s12672-023-00770-8

**Published:** 2023-08-19

**Authors:** Yujuan Zhao, Renhong Zhu, Yashu Wang, Keqiang Wang

**Affiliations:** 1 Comprehensive Ward, Yingsheng Hospital District of The Affiliated Tai’an City Central Hospital of Qingdao University, Tai’an, China; 2 Department of Laboratory Medicine, Tai’an Tumor Prevention and Treatment Hospital, Tai’an, China; 3 Department of Laboratory Medicine, The Affiliated Tai’an City Central Hospital of Qingdao University, Tai’an, China; 4 Department of Laboratory Medicine, Second Affiliated Hospital of Shandong First Medical University, Tai’an, China

**Keywords:** γδT cells, Cell classification, Biological function, Immunological function, Glioblastoma

## Abstract

Human peripheral blood T lymphocytes are classified into alpha–beta T (αβΤ) cells and gamma–delta T (γδΤ) cells based on the difference in T cell receptors (TCRs). αβT cells are crucial for the acquired immune response, while γδΤ cells, though only a small subset, can recognize antigenic substances. These antigens do not need to be processed and presented and are not restricted by MHC. This distinguishes γδΤ cells from αβT cells and highlights their distinct role in innate immunity. Despite their small number, γδΤ cells hold significant significance in anti-tumor, anti-infection and immune regulation. Glioblastoma (GBM) represents one of the most prevalent malignant tumors within the central nervous system (CNS). Surgical resection alone proves to be an ineffective method for curing this type of cancer. Even with the combination of surgical resection, radiotherapy, and chemotherapy, the prognosis of some individuals with glioblastoma is still poor, and the recurrence rate is high. In this research, the classification, biological, and immunological functions of γδT cells and their research progress in anti-glioblastoma were reviewed.

## Introduction

Human peripheral blood T lymphocytes are classified into alpha–beta T (αβΤ) cells and gamma–delta T (γδΤ) cells based on the difference in T cell receptors (TCRs). αβΤ cells are vital for the acquired immune response. Human γδΤ cells were discovered in the 1980s. Given their distribution and absence of MHC (major histocompatibility complex) restriction in their immune response, human γδΤ cells serve a distinct role in innate immunity. γδΤ cells are made up of γ and δ chains, and they originate from the thymus. However, the peripheral tissues and organs are mature, accounting for about 0.5% of lymphocytes in the peripheral blood of healthy adults [1–3]. Recent research has demonstrated that γδ cells are crucial for anti-tumor, anti-infection, and immune regulation [4–12]. Glioblastoma (GBM) is one of the most prevalent malignant tumors in the central nervous system (CNS). The treatment of glioblastoma is particularly challenging, and surgical resection alone is rarely curative. Despite the combination of surgical resection, radiotherapy, and chemotherapy, the prognosis remains unfavorable for some patients, with a high rate of recurrence [13]. In the 2021 World Health Organization (WHO) classification of CNS tumors, low-grade gliomas (LGG) encompassed grades 1 and 2, while high-grade gliomas (HGG), which included certain types of CNS gliomas, were categorized into grades 3 and 4. Glioblastoma multiforme (GBM), classified as WHO grade 4, represents the most invasive and malignant primary brain tumor, with a mere 5% survival rate over 5 years [14]. Therefore, it is crucial to develop innovative strategies to effectively treat gliomas and significantly reduce mortality rates. The current article provides a review of the classification, biological and immunological functions of γδΤ cells, the expression characteristics of γδΤ cells in patients with GBM, and the progress of these cells against GBM.

## Classification of γδT cells

### Structural classification of γδT cells

**Fig. 1:**
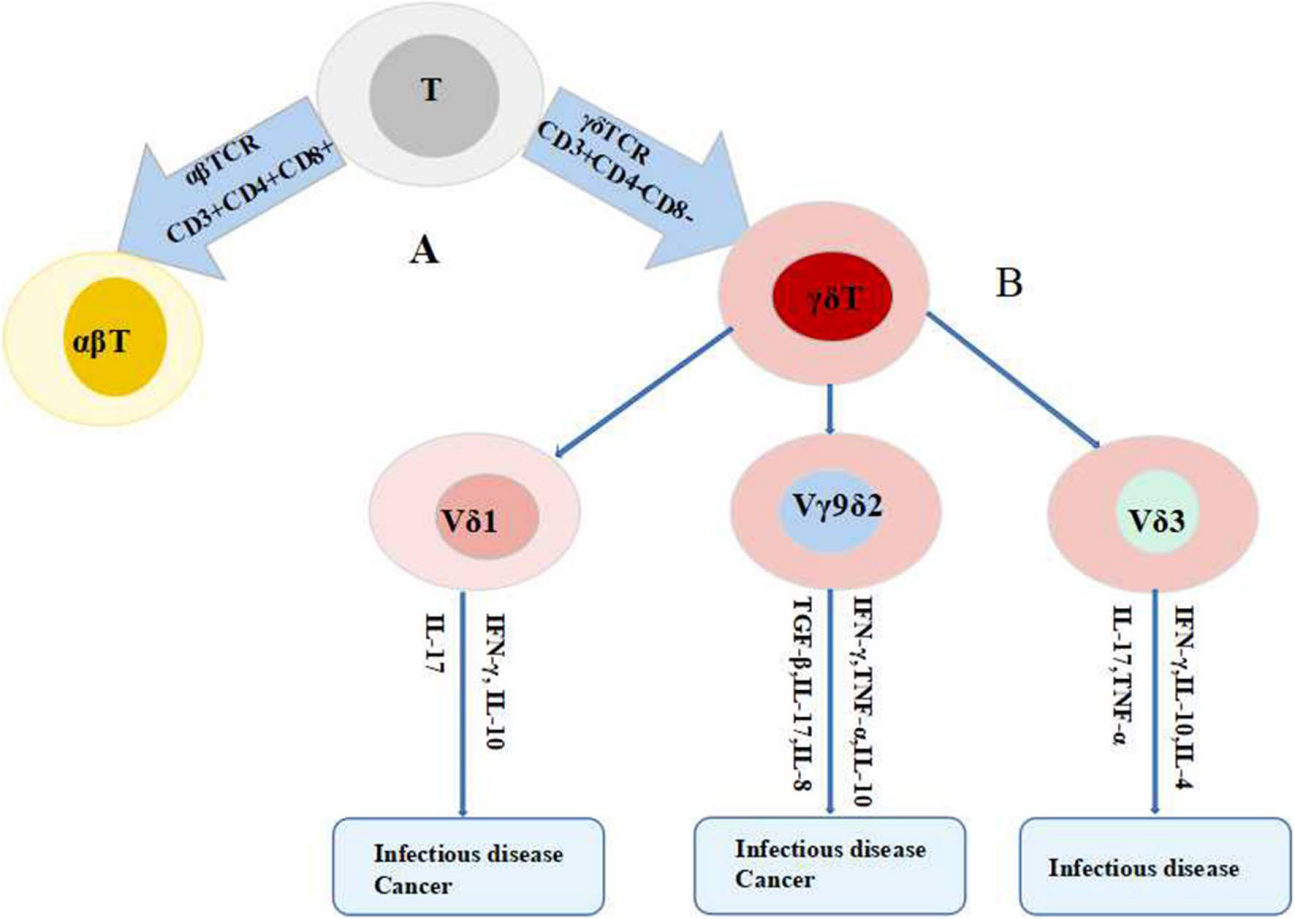
Classification and characteristics of human γδT cell subsets. **A** T cells are classified into αβT cells and γδT cells according to the differences in the types of their cell receptors (T cell receptor, TCR). **B** γδT cells can be divided into Vδ1γδT cells, Vδ2γδT cells and Vδ3γδT cells according to the difference of their δ chains. They play an important role in infectious disease and/or cancer

Regulation of the delta chain of human γδT cells is carried out by three Vδ genes (1–3), which leads to their classification into Vδ1γδT cells, Vδ2γδT cells and Vδ3γδT cells based on the variation in their delta chains (Fig. 1) [15].

1. Vδ1γδT cells: Vδ1γδT cells are primarily found in the thymus, mucosa, and subcutaneous tissues, representing the most abundant subgroup present on the mucosal surface. This subgroup is crucial for maintaining the integrity of epithelial tissue. Moreover, it also secretes perforin and granzyme by producing interferon-γ (IFN-γ), IL-10, and small amounts of IL-4, IL-2, and other cytokines. These chemical substances, along with the secretion and expression of chemokines, exert a cytotoxic effect, thereby participating in the anti-tumor response. Moreover, this subgroup has an inhibitory effect on a variety of epithelial-derived tumors and certain leukemias. Vδ1γδT cells can participate in the resistance to microbial infections by secreting IL-17 and the pro-inflammatory cytokine IFN-γ. Vδ1γδT cells express the helper stimulator CD8 on the cell surface, playing an essential role in activating helper T cells. The mucosa and epithelial tissues are the first barrier against pathogen invasion, and they are also common sites for tumor development. The high proportion of γδT cells in these tissues suggests their crucial role in tumor immunity, as well as in protection against microbes and parasites [3, 8, 16].
2. Vδ2γδT cells: Vδ2γδT cells are primarily found in peripheral blood. During the TCRγδ recombination process, the Vδ2 chain almost exclusively combines with Vγ9, resulting in the formation of Vγ9Vδ2T cells [17]. Vγ9Vδ2T cells, being the predominant circulating cells, comprise 0.5% to 5% of adult peripheral blood. These cells can be specifically activated by phosphorylated antigens that are produced either by microorganisms or by abnormally transformed cells, causing exogenous infections and endogenous abnormal cell transformations [16, 18]. According to the different surface markers of Vγ9Vδ2γδT cells, they can be classified into four subgroups: CD45RA+CD27+ naive cells, CD45RA−CD27+ central memory cells, CD45RA−CD27-effector memory cells, and CD45RA+CD27-terminally differentiated cells. The first two types of cells are primarily located in secondary lymphoid tissues and proliferate under the stimulation of isopentenyl pyrophosphate. However, they typically do not exert direct effector functions. On the other hand, the last two types of cells are mainly distributed in infection and tumor sites, performing direct effector functions such as secretion of cytokine IFN-γ and tumor necrosis factor-α (TNF-α), as well as cytotoxicity [19].
3. Vδ3γδT cells: Vδ3γδT cells are abundant in the liver and are the least abundant subgroup in the body, accounting for only 0.2% of circulating γδT cells. CD56, CD161, and NK cell surface activation receptor D are expressed on their surface. Studies have shown that Vδ3γδT cells can not only secrete IFN-γ, TNF-α, and IL-4 to enhance the immune function of the body but also enhance the recognition of CD1d to act on CD1d+ target cells and induce dendrites. Cells (DCs) are transformed into antigen-presenting cells (APCs), and they are constantly detected and identified as cancerous cells [20, 21].

### Functional classification of γδT cells

The structural heterogeneity among γδT cell subgroups leads to a wide range of functional diversity. As a result, based on their distinct functions, they can be classified into γδT cells that secrete IFN-γ (IFN-γ+γδT cells), γδT cells that secrete IL-17 (γδT17 cells), and regulatory γδT cells (γδTreg cells) [22], among others.

1. IFN-γ+γδT cells: IFN-γ+γδT cells are a type of γδT cells that highly express IFN-γ, which undergo functional differentiation in the thymus. Various factors in the thymus microenvironment, such as γδTCR and transforming growth factor β receptors, lymphotoxin β receptors, CD2, skint-1, intracellular molecule B lymphokinase, and promyelocytic leukemia zinc finger genes are all involved in this process [23]. IFN-γ+γδT cells play a crucial role in autoimmune diseases, tumor surveillance, host defense, and incision healing. Studies have found that their number in hepatitis B patients has increased significantly, suggesting functional IFN-γ+γδT cells also play an important role in controlling infection caused by the hepatitis B virus [24].
2. γδT17 cells: γδT17 cells belong to the subgroup of Vδ1γδT cells derived from thymus, which mainly secrete IL-17. They are capable of expressing aryl hydrocarbon receptors, retinoic acid-related nuclear orphan receptors γt, and IL-12 receptors such as Th17 cells, as well as CCR6 receptors. They can also directly act on pathogens through Toll-like receptors [25]. Among them, γδT17 cells with a terminally differentiated phenotype of CD27−CD45 RA+ can express tumor necrosis factor-related apoptosis-inducing ligands, granzyme B, FasL, and CD161. However, they do not produce IL-22 and IFN-γ. In terms of antigen activation, γδT17 cells can quickly trigger IL-8-mediated neutrophil migration and phagocytosis. Additionally, epithelial cells rely on IL-17 for the production of β defensins [18]. IL-17A produced by γδT17 cells also holds significant significance in the infection caused by the Mycobacterium BCG vaccine in the lungs, as well as in the development of granulomatous immune response induced by the BCG vaccine [26]. The above studies show that γδT17 cells play an important role in inflammation caused by microorganisms. Furthermore, γδT17 cells have been found to exert tumor-promoting effects. The IL-17 secreted by these cells can induce tumor angiogenesis. Furthermore, tumor-infiltrated γδT17 cells secrete IL-17, IL-8, TNF, and GM-CSF, which promote the proliferation of PMN-MDSC, forming an immunosuppressive microenvironment, thereby promoting tumor growth [27–29].
3. γδTreg cells: γδTreg cells mainly belong to the Vδ1 subgroup, with the Vδ1+CD27+CD25+ phenotype, and can express Foxp3 similar to the classic CD4 Treg cells. They mainly exert their inhibitory effect on the proliferation of CD4+ T cells through direct cell–cell contact. The cytokines secreted by γδTreg cells are mainly granulocyte–macrophage colony-stimulating factors and IFN-γ [30]. Moreover, γδTreg cells have a crucial role in various aspects such as anti-infection mechanisms, tumor immunotherapy, and graft-versus-host disease, among others. They exert these effects by regulating both innate and adaptive immune responses [31, 32].

## The function of γδΤ cells

### Biological function of γδΤ cells

**Fig. 2:**
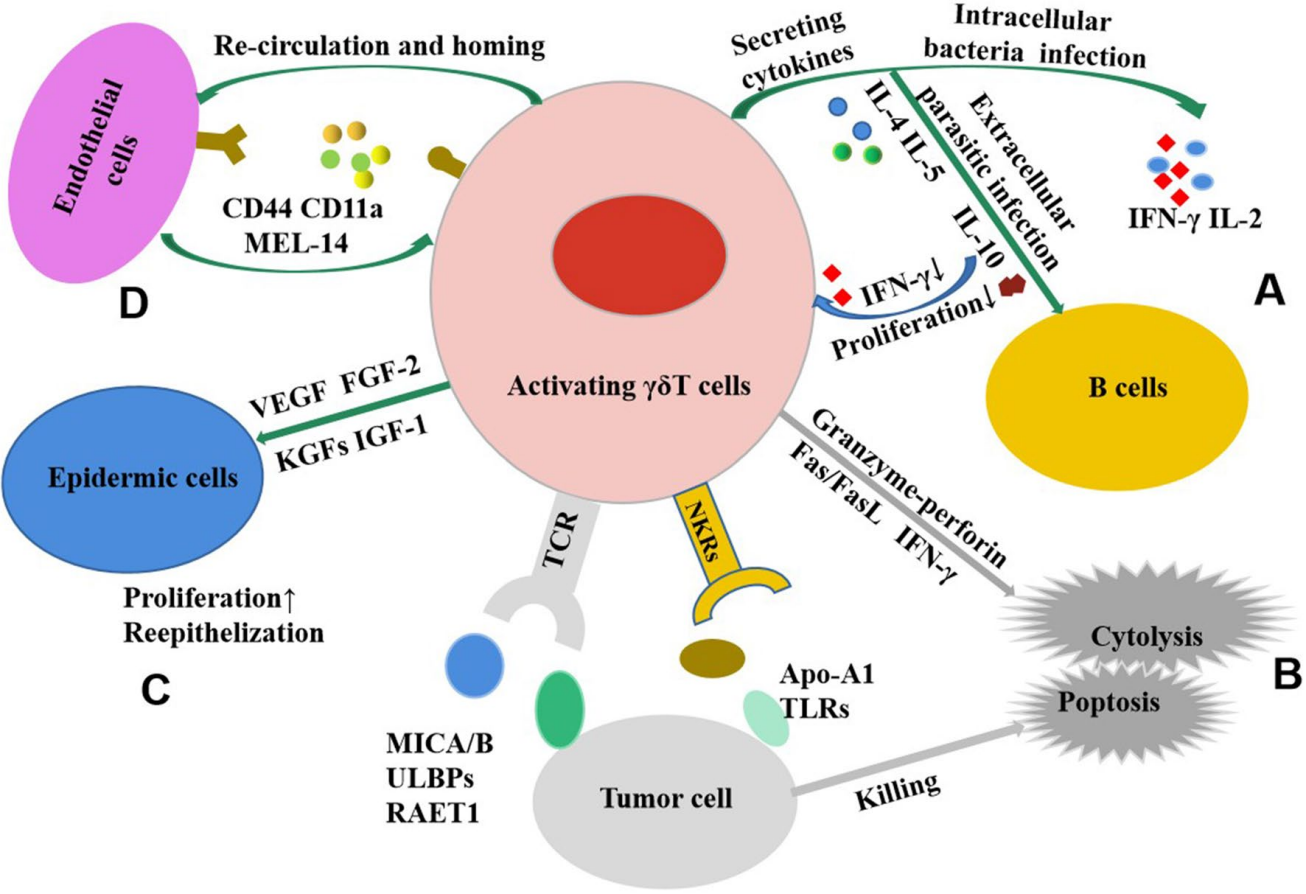
The biological function of γδΤ cells. **A** Cytokine production. During infection, γδΤ cells can exhibiting Th1-like or Th2-like effects; in turn, the IL-10 can inhibit the proliferation and secretion of γδΤ cells. **B** γδΤ cells recognize and kill tumor cells through TCR and NKG2D receptors, or direct lysis of target cells. **C** Promoting wound healing: γδΤ cells stimulate the proliferation of epidermal cells and the re-epithelialization of wounds by expressing VEGF,FGF-2, KGFs and IGF-1. **D** γδΤ cells bind to specific receptor molecules on endothelial cells using CD44, CD11a (LFA21) and MEL-14, thus mediating their recirculation and homing

Activated γδΤ cells exhibit various biological functions. Some of their notable functions include:

1. Cytokine production [33]: During intracellular bacterial infection, γδΤ cells have the ability to produce interferon-gamma (IFN-γ) and interleukin 2 (IL-2), exhibiting Th1-like effects similar to helper T lymphocyte type 1 cells. On the other hand, when infected by extracellular parasites, γδΤ cells produce IL-4, IL-5, and IL-10, which stimulate B cells and exhibit Th2-like effects similar to helper T lymphocyte type 2 cells. Additionally, the IL-10 produced during the aforementioned process can, in turn, inhibit the proliferation and secretion of cytokine IFN-γ by γδΤ cells [34].
2. Direct lysis of target cells: Activated γδΤ cells possess the ability to directly cleave target cells via the granzyme-perforin pathway. Moreover, they can trigger apoptosis of the target cells through Fas-FasL (transmembrane protein/transmembrane protein cytokines) and IFN-γ [35].
3. Recognition and killing of tumor cells: γδΤ cells are capable of recognizing stress-inducing molecules such as MICA, MICB, ULBP, and RAET1. Moreover, they can also recognize ectopic apolipoprotein A1 and Toll-like receptors present on the tumor surface [36]. MICA/B and ULBPs were expressed in various types of tumor epithelial cells. γδΤ cells, much like NK cells, recognize tumor cells unrestrictedly through NKG2D receptors. This suggests that even without the presence of human leukocyte antigen or tumor antigen, γδΤ cells retain their ability to eliminate target cells [37]. New immunotherapy strategies, such as chimeric antigen receptor (CAR) engineered γδT cells, can improve the efficacy of CAR-T cells, enhance anti-tumor effect and reduce its side effects [38–41].
4. Promoting wound healing: γδΤ cells are capable of responding rapidly to skin damage, and an increased presence of these cells can be observed at the wound site at 4 h [42]. A small quantity of vascular endothelial growth factor and fibroblast growth factor 2 were produced [43]. Activated γδΤ cells stimulate the proliferation of epidermal cells and the re-epithelialization of wounds by expressing KGFs and IGF-1 [44]. They also have the capacity to repair intestinal injury [45].
5. Mediate its recycling and homing: γδΤ cells, much like αβΤ cells, can bind to specific receptor molecules on endothelial cells using CD44, CD11a (LFA21) and MEL-14 (mouse CD62L APC labeled fluorescent monoclonal antibody). This binding facilitates γδΤ cells to adhere to endothelial cells, thus mediating their recirculation and homing (Fig. 2) [10].

### Immunological function of γδΤ cells

**Fig. 3:**
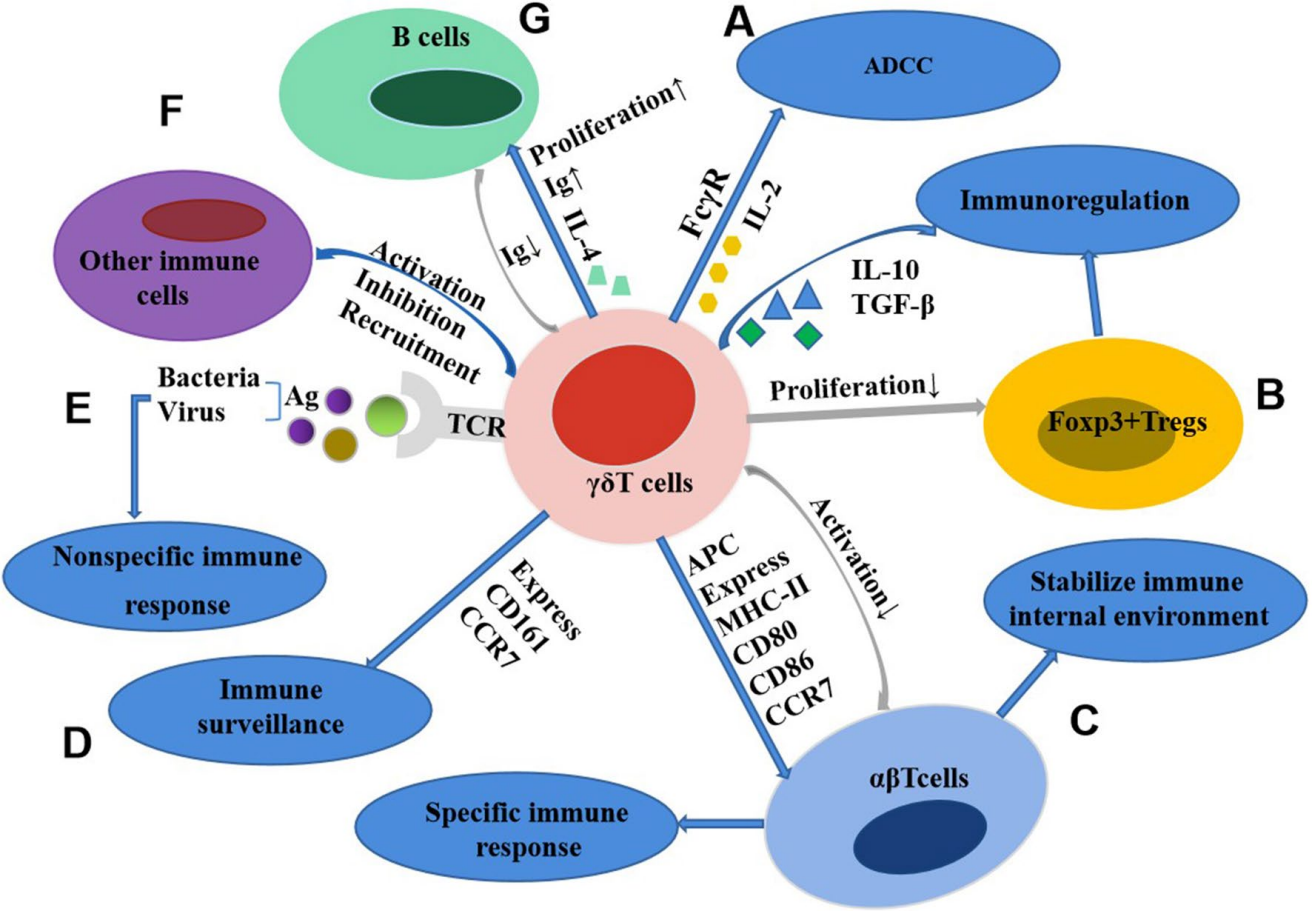
Immunological function of γδT cells. **A** Antibody-dependent cytotoxicity; **B** immunomodulatory function; **C** antigen presentation; stabilization of the internal immune environment; **D** immunesurveillance; **E** non-specific immune response; **F** γδΤ cells play their immunological roles by activating, inhibiting, or recruiting other immune cells. **G **Bidirectional action on B cells

Activated γδΤ cells perform a wide range of immunological functions:

1. Antigen presentation: Partially activated γδΤ cells can differentiate into antigen-presenting cells (APCs) and show high expression levels of MHC-class II molecules and CD80, CD86, and CCR7 (chemokine receptors) on their surface. Moreover, they can process antigens and present them to αβΤ cells, triggering a specific immune response [46].
2. Non-specific immune response: In the absence of APCs, γδΤ cells can be directly activated via their TCR for recognizing a variety of antigenic components of bacteria and viruses. This process plays a significant role in non-specific immune responses [7].
3. Immune surveillance: Memory γδΤ cells can prevent the spread of viruses, combat opportunistic infections, and perform immune surveillance by over-expressing CCR7 and CD161 on their surface [47]. Cytomegalovirus (CMV) infection is usually associated with the development of GBM [48]. Human non-Vδ2T cells can directly bind endothelial protein C receptor (EPCR), which is a MHC-like molecule similar to antigen presentation molecule CD1d and can bind to lipid. Adrenergic receptor A2 (EphA2) is a stress-related molecule that also participates in the activation of non-Vδ2T cells. Both EPCR and EphA2 are expressed on endothelial cells infected by CMV and up-regulated during the development of GBM tumor [49, 50]. GBM tumor cells express BTN-like protein BTN3A, which mediates the recognition of PAg by γδTCR and contributes to the antigenic response of Vγ9Vδ2 T cells [51, 52].
4. Immunomodulatory function: Activated γδΤ cells have the ability to suppress the proliferation of Foxp3+Tregs (regulatory T cells) [53]. They can also generate IL-10 and TGF-β (transforming growth factor β) to perform an immunomodulatory function [54].
5. Stabilization of the internal immune environment: γδΤ cells can inhibit the overactivation of αβΤ cells, thus maintaining the relative balance between αβΤ and γδΤ cells [55].
6. Antibody-dependent cytotoxicity: Certain membrane receptors, such as FcγR (IgG Fc receptor), contribute to antibody-dependent cell-mediated cytotoxicity (ADCC) and enhance their cytotoxic effects through the secretion of IL-2 [56].
7. Bidirectional action on B cells: The majority of γδΤ cells are directly activated by antigens to produce IL-4, which in turn stimulates B cell proliferation and secretion of immunoglobulin (Ig). However, certain subsets of γδΤ cells suppress the production of Ig by B cells.
8. Immunological function: γδΤ cells play their immunological roles by activating, inhibiting, or recruiting other immune cells. Their interactions with immune cells, including dendritic cells, granulocytes, macrophages, Langerhans cells, αβΤ cells, and B cells, are closely related to their anti-infective function (Fig. 3) [57].

## Characteristics of γδΤ cell expression in patients with GBM

**Fig. 4:**
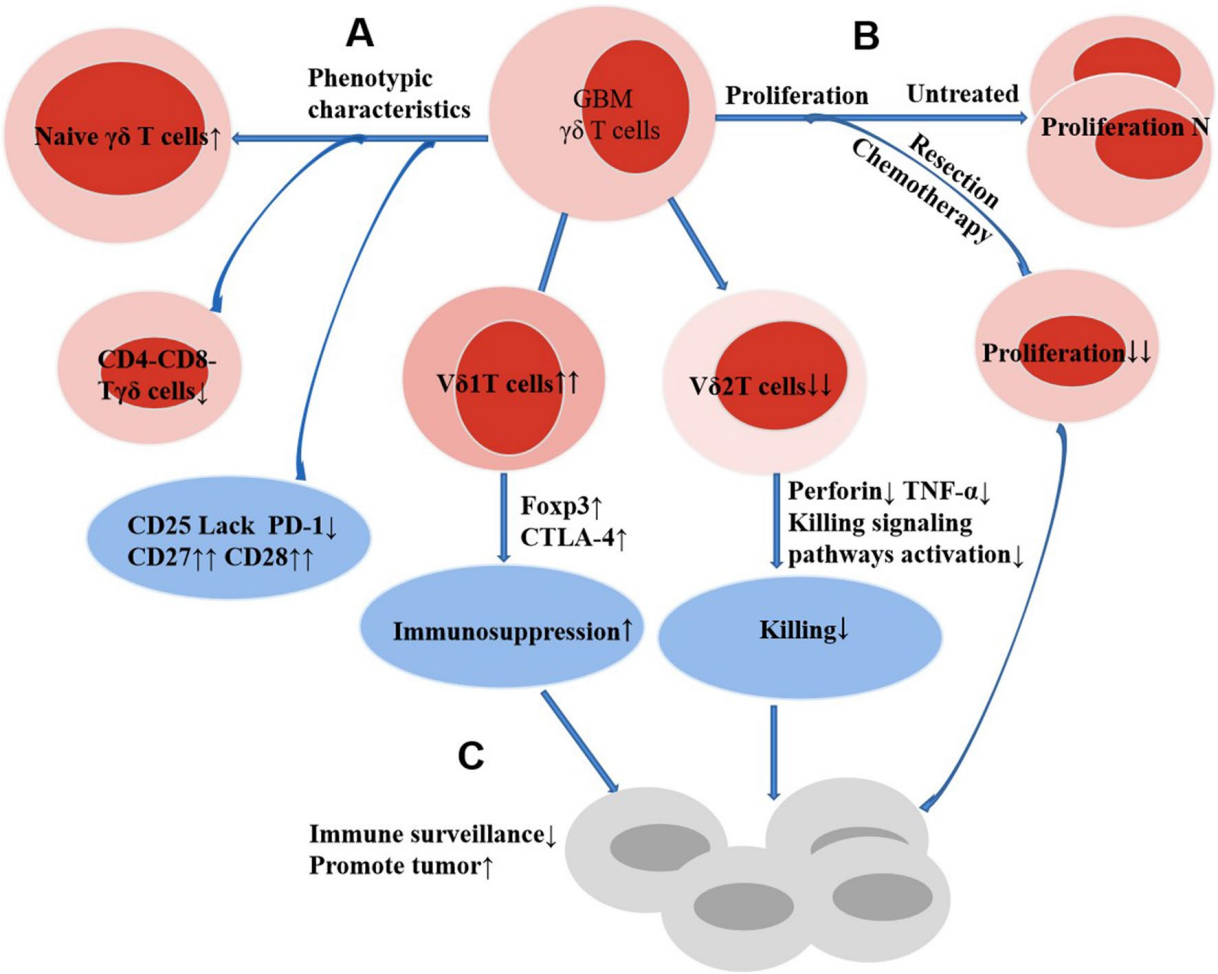
Characteristics of γδΤ cells in the peripheral blood of patients with GBM. **A** Phenotypic characteristics, a decrease in CD4−CD8−T γδ cells, CD25 and CD279 (PD-1); a significant increase in CD27 and CD28; an increase in immature γδΤ cells. **B** In terms of proliferation, γδΤ cells of untreated GBM patients have strong proliferative ability, while the proliferative ability of γδΤ cells decreased significantly after tumor resection or chemotherapy. **C** The peripheral blood γδΤ cells of GBM displayed characteristics of cell depletion, functional impairment, reduced proliferation ability, and an imbalance between Vδ1T cells and Vδ2T cell, thus promoting the occurrence and development of tumors

The proportion of total γδΤ cells in the peripheral blood of individuals with GBM was found to be similar to that of healthy individuals, but the absolute count showed a decreasing trend. Specifically, there was a decrease in double negative (CD4−CD8−) T γδ cells, an increase in immature γδΤ cells, a decrease in the expression levels of CD25 and CD279 (PD-1), and a significant increase in the expression levels of costimulatory markers CD27 and CD28 [58]. The balance between the two primary subsets, Vδ1 T cells to Vδ2 T cells, was disrupted. In the peripheral blood of individuals with GBM, Vδ1 T cells became the dominant subset of γδΤ cells. In individuals with GBM, there was a substantial increase in the proportion of Vδ1T cells, the expression of molecules associated with immunosuppression (Foxp3, CTLA-4), and immunosuppressive function. Conversely, the proportion of Vδ2T cells, the expression of perforin and TNF-α, and the activation of cytotoxicity-related signal pathways considerably decreased. Consequently, the lethality significantly decreased in these individuals. In terms of proliferation, γδΤ cells of untreated GBM patients still had a strong proliferative ability, while the proliferative ability of γδΤ cells decreased significantly after tumor resection or chemotherapy. Compared to healthy people, γδΤ cells in the peripheral blood of people with GBM displayed characteristics of cell depletion, functional impairment, reduced proliferation ability, and an imbalance between Vδ1T cells and Vδ2T cells. These characteristics might contribute to immunosuppression and enable tumors to evade immune surveillance, thus promoting the occurrence and development of tumors [59, 60]. Different researchers have different opinions on GBM-infiltrating γδΤ cells. Lee et al. found that γδΤ cells infiltrated in tumors, mainly Vγ9Vδ2T cell subtypes, and unique Vγ9Vδ2T cells controlled by Vγ9vγ2 sequence gave priority to infiltrating GBM. GBM infiltrating γδΤ cells exhibit high plasticity. Their activity is closely related to the activity of cytotoxic T lymphocytes and regulatory T cells, showing anti-tumor or pro-tumor activity. These findings, together with other studies, have confirmed that γδΤ cells can exhibit different phenotypes according to the surrounding microenvironment, including Th1 type, Th2 type, Th17 type, follicular Th2 type, or Treg characteristics [61–64]. However, Bryant et al. [60] found no infiltration of γδΤ cells in the tumor parenchyma. The emergence of these two different research outcomes may be linked to the timing of specimen selection, subtle differences in research methods, and other influencing factors. To sum up, γδT cells in peripheral blood of GBM patients are characterized by imbalance of Vδ1T cells and Vδ2T cells, decrease of cell killing function and proliferation ability, but activated GBM patients γδT cells still have cytotoxicity and dissolve GBM tumor cells in vitro [60]. GBM tumor infiltrating γδT cells have high plasticity. Their existence may be strongly associated with the onset and progression of gliomas (Table 1, Fig. 4).

**Table 1:** Characteristics of γδΤ cell expression in patients with GBM

Year	Author	Patients	Sample type	Method	Results
2022	Belghali [58]	Initially enrolled GBM Patients	Peripheral blood	Flow cytometry	γδT cells(N), Vδ2 ↓, Vδ1↑, CD4−CD8−Tγδ cells↓, Naive γδT cells↑, CD25(−), CD279(PD-1)↓, CD27, CD28↑
2018	Yue [59]	Undergoing glioma resection, unreceived chemotherapy and radiotherapy before surgery	Peripheral blood	Flow cytometry Western blot assay CFSE proliferation assay	Ratio of Vδ2 T cells↓↓, ratio of Vδ1 T cells↑↑, Foxp3+ Vδ1T cells↑↑, CTLA-4+ Vδ1Tcells↑, Perforin+ Vδ2T cells ↓↓, TNF-α+ Vδ2T cells↓↓
2009	Bryant [60]	Presenting with CT or MRI evidence of probable GBM, enrolled following histological diagnosis	Peripheral blood	Flow cytometry Modified mitogen proliferation assay	Ratio of Vδ2 T cells↓↓, ratio of Vδ1 T cells↑↑, Foxp3+ Vδ1T cells↑↑, CTLA-4+ Vδ1Tcells↑, Perforin+ Vδ2T cells ↓↓, TNF-α+ Vδ2T cells↓↓
2009	Bryant [60]	Presenting with CT or MRI evidence of probable GBM, enrolled following histological diagnosis, had partial resection	FFPE	IHC: staining for CD3 and TCR γδ	No evidence for infiltration of CD3^+^ cells or TCR-γδ^+^ cells deep within the tumor parenchyma
2019	Lee [61]	Post-adjuvant treatment GBM patients	Tumor tissues	TCRS; IHC: staining for TCRγδ	γδΤ cells infiltrated in tumors, mainly Vγ9Vδ2T cell subtypes, and unique Vγ9Vδ2T cells controlled by Vγ9vγ2 sequence gave priority to infiltrating GBM

N: normal; ↓: decrease; ↑: increase; (−): negative; ↑↑: significantly increase; ↓↓: significantly decrease; FFPE: formalin-fixed, paraffin-embedded; TAγδTCC: total absolute γδT-cell counts; Pre-op: newly diagnosed GBM patients; EPS: early postoperative stages; ACTS: after cytoreductive therapy stages; IHC: immunohistochemistry; TCRS: T-cell receptor (TCR) sequencing

## Anti-GBM effect of γδΤ cells

Numerous reports have highlighted that γδΤ cells exhibit certain cytotoxic effects on GBM, although these effects vary across GBM cell lines. γδΤ cells display cytotoxicity towards GBM cell lines U87, U138, T70, U373, U251. Moreover, local injection of expanded γδΤ cells in vitro can slow down the tumor progression and improve the survival rate of human U251MG tumor xenografted non-thymic nude mice. However, it showed almost no cytotoxic effect on A172 cells. This difference might be associated with the expression of MICA/B, UL-16 binding protein (ULBP), intercellular adhesion molecule (ICAM-1), and PVR on the surface of tumor cells [62, 65, 66]. γδΤ cells have a broad capacity to recognize and immediately respond to various MHC-like stress-induced autoantigens. The majority of these autoantigens exhibit expression in human GBM cells but not in adjacent normal brain tissues [67–69]. When the expanded γδΤ cells were co-cultured with glioma cells, γδΤ cells recognized the related antigens expressed on the tumor cell surface through their surface TCR or natural killer receptor NKG2D and differentiated memory cells. These γδΤ cells then induced the tumor cells to undergo apoptosis by releasing substances such as perforin and granzyme B and by secreting Th1 cytokines IFN-γ and TNF-α [66, 70–75]. These provide a theoretical basis for adoptive immunotherapy of GBM with γδT cells [52, 76]. Nonetheless, the ability of γδΤ cells to suppress GBM tumor cells is limited and occurs in a dose-dependent manner [60, 66]. Research has demonstrated that nitrogen-containing phosphonates, including zoledronic acid (ZOL), minopronate (MDA), and chemotherapeutic drugs, can effectively improve the anti-GBM activity of γδΤ cells. ZOL and MDA can not only directly induce apoptosis of glioma cells but also enhance the production of IFN-γ and TNF-α by γδΤ cells and lead to the accumulation of intracellular IPP by interfering with the metabolic pathway of methoxylphosphonate. Additionally, γδΤ cells recognize and kill these cells containing phosphonate antigens through TCR γδ receptors [9, 18, 77].

Low-dose ZOL treatment not only significantly increased the cytotoxicity of γδΤ cells to GBM-sensitive strains but also strongly triggered the killing of γδΤ cells to resistant strain A172 cells. γδΤ cells recognized GBM cells that had been pretreated with ZOL using specific membrane surface receptors, and they killed these cells through a direct cytotoxicity mechanism. This enhanced cell-killing effect may be mediated by the expression of PVR on GBM cells and the existence of NK cell-activated receptor molecule (DNAM-1) on γδΤ cells [65]. Jarry et al. further confirmed the sensitizing effect of ZOL on GBM cells. Using ^51^Cr release assay, it was found that allogeneic Vγ9Vδ2T cells had no natural response to U-87MG cells and primary GBM-10 cells, while zoledronate pretreatment of GBM cells triggered significant dose-dependent antigen activation of Vγ9Vδ2T cells [51]. By stereotactic administration, they found that zoledronate or Vγ9Vδ2T cells alone did not significantly increase the median survival time of orthotopic implanted U-87MG or BMG-10 NSG mice. However, single and double administration of zoledronate and Vγ9Vδ2T cells significantly increased the survival rate of mice [51]. Primary GBM-10 is a kind of tumor cells that express high-level “stemness” markers CD133, CD90 and CD44, which are disseminated and invasive, and can reproduce the physiological characteristics of human GBM. The above results show that stereotactic administration of allogeneic human Vγ9Vδ2T cells combined with zoledronate can effectively eliminate not only low invasive tumors but also heterogeneous primary human GBM tumors characterized by “stemness” and invasive [51, 78].

The combination of MDA and γδΤ cells not only effectively induced the apoptosis of GBM cells in vitro but also significantly inhibited the growth of U87MG-derived tumors in NOG mice in vivo. Nakazawa et al. implanted U87MG cells subcutaneously into high immunodeficiency (NOG) mice and injected MDA/GDT intraperitoneally. It was found that MDA combined with GDT could inhibit the growth of unestablished U87MG-derived subcutaneous tumors, and NOG mice had good tolerance to systemic MDA/GDT therapy [75]. γδΤ cells are activated by TCR to recognize IPP metabolites in GBM cells exposed to MDA and induce apoptosis by releasing granzyme B and TNF-α in a cysteine protease (caspase) dependent manner. Therefore, the combination of ZOL or MDA and γδΤ cells produced in vitro may be an effective treatment for patients with GBM [51, 65, 74, 75].

IL-21, a nodular cytokine, is a sensitizing factor of Vγ9Vδ2T cells. It enhances their cytolytic activity by elevating the levels of granzyme B within Vγ9Vδ2T cells. The sensitization of IL-21 can last for at least 24 h in the absence of this factor, and does not affect the migration rate of Vγ9Vδ2T cells in vivo [79]. Joalland et al. established an invasive in situ GBM mouse model by stereotactic implantation of GBM-1 cells into NSG mice. After stereotactic administration, it was found that IL-21-sensitized Vγ9Vδ2T cells could eradicate GBM and significantly improve the survival rate of mice. These results show that IL-21-sensitized allogeneic Vγ9Vδ2T cells have natural cytotoxicity to heterogeneous invasive primary human GBM tumors [79].

**Fig. 5:**
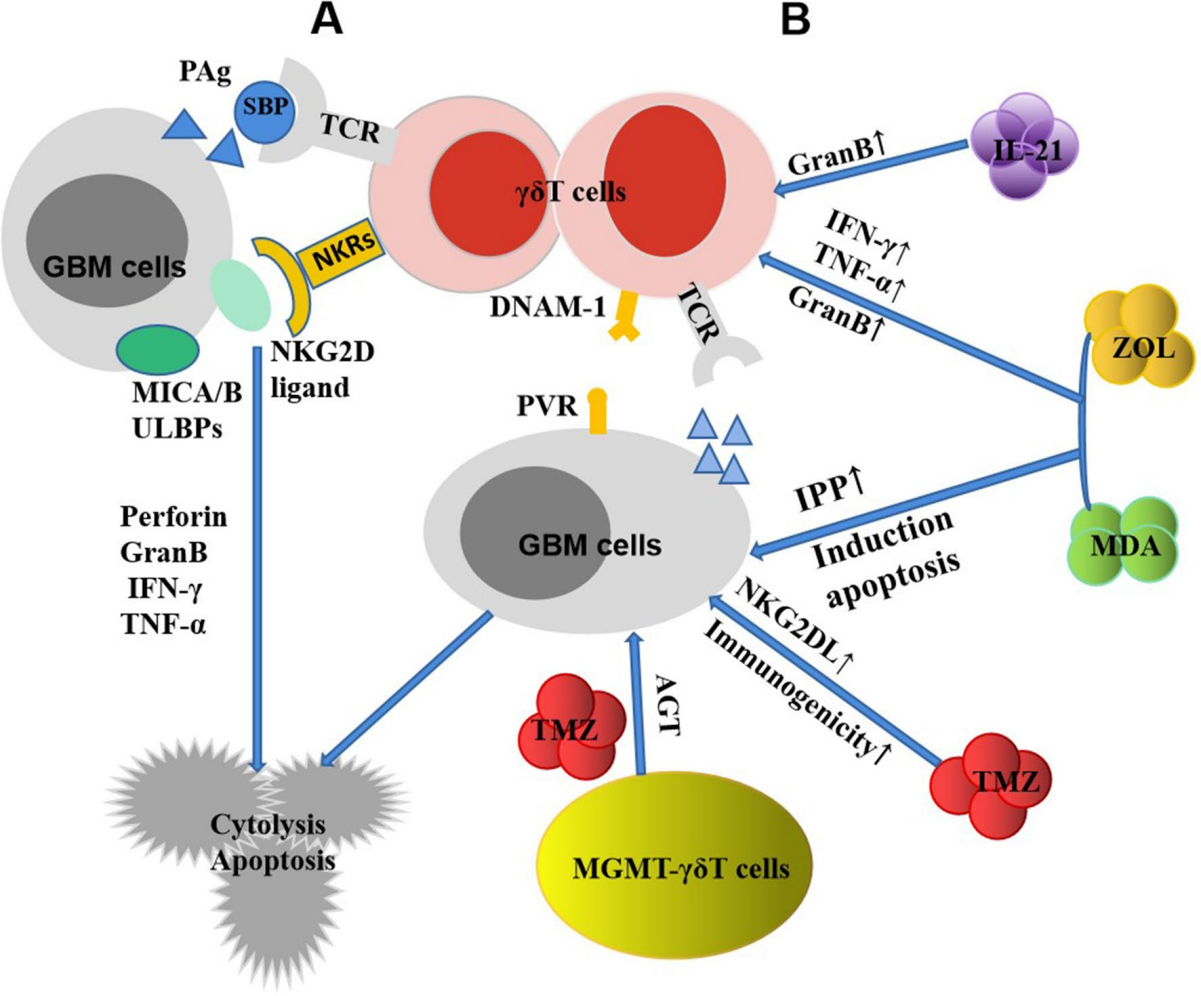
Killing effect of γδΤ cells on glioma cells. **A** γδΤ cells recognized the related antigens expressed on GBM cell surface through their surface TCR or NKG2D and differentiated memory cells. These γδΤ cells then induced the tumor cells to undergo apoptosis by releasing substances such as perforin and granzyme B and by secreting Th1 cytokines IFN-γ and TNF-α. **B** IL-21, ZOL, MDA and chemotherapeutic drugs, can effectively improve the anti-GBM activity of γδΤ cells

Temozolomide (TMZ) is the main chemotherapeutic drug used in the treatment of GBM. It can temporarily upregulate a variety of emergency-induced NKG2D ligands, improve the immunogenicity of GBM, and make GBM cells sensitive to γδΤ cell-mediated lysis [80]. NKG2D ligand was also expressed in glioma stem cells, and its expression was significantly upregulated under the stimulation of TMZ [81]. However, TMZ also has a high cytotoxic effect on γδΤ cells. γδΤ cells modified by methylguanine DNA methyltransferase (MGMT) produce O6-alkylguanine DNA alkyltransferase (AGT), which allows γδΤ lymphocytes to play a role in the therapeutic concentration of TMZ and empowers them with resistance to TMZ. MGMT modified γδΤ cells were mainly effective memory phenotype, and gene modification did not change the proliferative ability and cytotoxicity of γδΤ cells. The combination of MGMT-modified γδΤ cells and TMZ can effectively improve the survival rate of primary GBM tumor xenotransplantation mice [82, 83]. γδΤ cells are genetically modified to resist the toxicity of chemotherapeutic drugs in order to realize the combined application of chemotherapy and immunotherapy. INB-200 is a genetically modified autologous γδT cell immunotherapy developed by IN8bio for the treatment of glioblastoma (GBM). Currently, an ongoing phase I clinical trial (NCT04165941) is testing the safety and tolerability of this therapy in combination with temozolomide (TMZ) in patients with newly diagnosed glioblastoma. Chauvin et al. [84] reported that allogeneic human Vγ9Vδ2T cells possess the ability to spontaneously recognize and clear human GBM mesenchymal cells without any treatment and significantly prolong the life span of tumor-bearing mice. This effect is mediated by γδΤCR and regulated by the stress-related NKG2D pathway (Table2, Fig. 5).

**Table 2:** Preclinical trials of the anti-GBM based on γδT cells

Year	Author	Effector cells	Tumor type	Sensitization factor	Results
2014	Nakazawa[65]	Allogeneic γδT cells	U87MG, U138MG and A172	ZOL	Kill GBM cell lines
2016	Jarry [51]	Allogeneic Vγ9Vδ2T cells	U87MG/BMG-10 NSG mice	ZOL	Efficiently eliminate tumor cells, strongly improved the survival of mice
2009	Bryant [60]	γδT-cells from patients/healthy volunteers	D54, U373, U251 and primary GBM	–	Kill D54, U373, and U251, as well as primary GBM, without cytotoxicity to primary astrocyte cultures
2011	Bryant [66]	Allogeneic Vγ9Vδ2T cells	New or established U251 AN mice	–	Significantly inhibit tumor progression and improve survival
2011	Cimini [74]	Allogeneic Vγ9Vδ2T cells	T70, U251, U373	ZOL	Vδ2 T-cell lines recognize glioma cell and differentiate into effector memory cells able to release Perforin, and kill glioma cells; ZOL enhanced the killing function
2016	Nakazawa [75]	Ex vivo expanded GDT	U87MG, U138MG; U87MG-NOG mice	MDA	MDA and GDT synergistically potentiated GDT-mediated growth inhibition of U87MG and U138MG cells; MDA elicited anti-GBM effects in synergy with GDT in vivo
2018	Joalland [79]	Allogeneic Vγ9Vδ2T cells	GBM-1-NSG-mice	IL-21	Eradicate GBM, improve the survival rate of GBM-1-NSG-mice
2016	Chitadze [80]	Allogeneic Vγ9Vδ2T cells	A172, T98G, U87MG and U251MG	TMZ	TMZ increased the expression of NKG2DLs; moderately affecting ULBP2 shedding; facilitate Vγ9Vδ2T cell-mediated GBM cell killing
2013	Lamb [82]	MGMT-modified γδT-cells	U87MG, U373^TMZ-R^, and SNB-19^TMZ-R^	TMZ	Cytotoxicity to U87MG, modified γδ T cells nearly equivalent to unmodified; significant killing to U373TMZ-R and SNB-19TMZ-R cells
2021	Lamb [83]	MGMT-modified γδT-cells	Primary GBM PDX mice	TMZ	DRI effective against primary high grade gliomas
2019	Chauvin [84]	Allogeneic Vγ9Vδ2 T cells	Human primary GBM, GBM-1/GBM-10-NSG mice	–	Spontaneously recognize and eliminate GBM-1; significantly increased GBM-bearing mouse lifespan

–: lack of sensitizing factor; ZOL: Zoledronate; AN: athymic nude； MDA: minodronate; GDT: γδT cell; NOG: NOD.Cg-Prkdc^scid^ Il2rg^tm1Sug^/Jic; NSG: NOD.Cg-Prkdcscid Il2rgtm1Wjl/SzJ; TMZ: Temozolomide; TMZR: TMZ resistant; DRI: drug resistant immunotherapy, combination therapy of TMZ and MGMT-modified γδ T cells; GBM-1: human mesenchymal tumor cells representative; GBM-10: human CNP tumor cells representative; CNP: classical, neural, and proneural subtypes

## Concluding remarks

To sum up, γδΤ cells have the ability to suppress and kill GBM cells. Consequently, immunotherapy strategies based on γδΤ cells could potentially become a novel approach for treating GBM. It might be worthwhile to consider the development of drugs that can expand, activate and promote the function of γδΤ cells in targeting GBM. In summary, increasing the number of γδΤ cells and enhancing their functioning within the GBM microenvironment is crucial for GBM treatment strategies that are based on γδΤ. It is believed that with the deepening of the study, γδT cells will achieve ideal results in anti-GBM therapy.

## Declarations

### Data availability

No data were involved.
